# Supplementary material for: Differential responses of the acidobacterial community in the topsoil and subsoil to fire disturbance in Pinus tabulaeformis stands
Source: PeerJ. 2019 Dec 11;7:e8047. doi: 10.7717/peerj.8047 (PMC6911345; doi:10.7717/peerj.8047)

**Differential Responses of the Acidobacterial Community in the Topsoil and Subsoil to Fire Disturbance in** ***Pinus tabulaeformis* Stands**

Weike Li^1^, Xiaodong Liu^1^, Shukui Niu^1^

^1^The College of Forestry, Beijing Forestry University, No. 35 Qinghua East Road, Haidian District, Beijing 100083, China.

Corresponding author:

Xiaodong Liu

The College of Forestry, Beijing Forestry University, No. 35 Qinghua East Road, Haidian District, Beijing 100083, China.

Email address: xd_liu@bjfu.edu.cn

Table S1 General situation of sampling plots

| Plots | Fire severity | Mean DBH/cm | Canopy density | Mean height/m | Mean scorched height/m | Tree mortality/% | Dominant species |
| --- | --- | --- | --- | --- | --- | --- | --- |
| HA | High severity | 22.30 | <0.1 | 12.42 | 12.42 | 100 | *Deutzia grandiflora, Lespedeza bicolor, Rhododendron micranthum*, *Setaria viridis,Viola acuminata* |
| HB |  | 27.10 | <0.1 | 11.05 | 11.05 | 100 |  |
| HC |  | 22.49 | <0.1 | 10.95 | 10.95 | 100 |  |
| MA | Moderate severity | 22.87 | 0.32 | 11.02 | 3.64 | 66.72 | *Pinus tabulaeformis, Spiraea pubescens, Deutzia grandiflora, Dendranthema chanetii, Carex duriuscula* |
| MB |  | 19.90 | 0.39 | 9.70 | 2.85 | 63.46 |  |
| MC |  | 22.10 | 0.28 | 10.80 | 3.12 | 54.60 |  |
| LA | Low severity | 24.33 | 0.56 | 11.99 | 1.47 | 16.35 | *Pinus tabulaeformis, Lespedeza bicolor, Spiraea pubescens, Dendranthema chanetii, Carex duriuscula* |
| LB |  | 21.80 | 0.59 | 9.90 | 1.66 | 18.20 |  |
| LC |  | 25.03 | 0.52 | 13.66 | 0.79 | 13.26 |  |
| CA | Control | 20.90 | 0.60 | 11.50 | 0 | 0 | *Pinus tabulaeformis, Spiraea pubescens, Lespedeza bicolor, Carex duriuscula*, *Ranunculus japonicus* |
| CB |  | 23.06 | 0.69 | 11.47 | 0 | 0 |  |
| CC |  | 21.17 | 0.65 | 10.40 | 0 | 0 |  |

Table S2 The f- and p-values for each diversity index of understory vegetation

|  | The diversity indices of the understory vegetation | | |
| --- | --- | --- | --- |
|  | H’Vegetation | D’Vegetation | J’Vegetation |
| f-values | 8.737 | 6.340 | 6.572 |
| p-values | 0.007 | 0.017 | 0.015 |

Table S3 The f- and p-values for sequencing results and acidobacterial α-diversity which are analysised by One-way ANOVA

|  | Sequencing results and acidobacterial α-diversity indices | | | | | | | |
| --- | --- | --- | --- | --- | --- | --- | --- | --- |
|  | shannon | | simpson | | otu | | reads | |
|  | T | S | T | S | T | S | T | S |
| f-values | 1.254 | 2.099 | 1.450 | 1.805 | 1.744 | 1.587 | 2.452 | 2.062 |
| p-values | 0.353 | 0.179 | 0.299 | 0.224 | 0.235 | 0.267 | 0.138 | 0.184 |

Table S4 The p-values for sequencing results and acidobacterial α-diversity which are analysised by independent-sample t-test

|  | Sequencing results and acidobacterial α-diversity indices | | | | |
| --- | --- | --- | --- | --- | --- |
|  |  | shannon | simpson | otu | reads |
| p-values | H | 0.001 | 0.007 | 0.433 | 0.010 |
|  | M | 0.564 | 0.074 | 0.662 | 0.908 |
|  | L | 0.230 | 0.268 | 0.142 | 0.160 |
|  | C | 0.197 | 0.208 | 0.406 | 0.953 |

Table S5 Relationships between the relative abundance of *Acidobacteria* subgroups (topsoil) and environment factors (topsoil) by Spearman's rank correlation coefficient test

|  | | Environmental factors | | | | | | | | |
| --- | --- | --- | --- | --- | --- | --- | --- | --- | --- | --- |
|  |  | TN | NH_4_^+^-N | pH | H’_Vegetation_ | D’_Vegetation_ | J’_Vegetation_ | DBH | Hight | Canopy density |
| *Acidobacteria* subgroups | Sub1 | -0.067 | 0.698* | -0.002 | -0.692* | -0.246 | -0.197 | 0.278 | 0.015 | 0.644* |
|  | Sub2 | 0.266 | 0.517 | -0.724** | -0.38 | -0.217 | -0.128 | -0.023 | 0.095 | 0.318 |
|  | Sub3 | 0.003 | 0.115 | -0.059 | -0.651* | 0.046 | -0.494 | -0.134 | 0.205 | 0.01 |
|  | Sub4 | 0.016 | -0.361 | -0.092 | 0.273 | 0.099 | 0.018 | -0.012 | -0.197 | -0.038 |
|  | Sub5 | -0.597* | -0.15 | 0.688* | -0.531 | 0.043 | 0.317 | 0.2 | -0.04 | -0.15 |
|  | Sub6 | 0.099 | -0.562 | 0.093 | 0.848** | 0.084 | -0.112 | -0.241 | 0.103 | 0.063 |
|  | Sub7 | -0.077 | -0.655* | 0.016 | -0.068 | 0.268 | -0.232 | -0.188 | 0.02 | -0.645* |
|  | Sub10 | -0.712** | -0.732** | 0.612* | -0.369 | -0.306 | 0.227 | -0.114 | -0.197 | -0.707** |
|  | Sub11 | -0.369 | -0.368 | 0.345 | -0.709** | -0.529 | 0.175 | -0.004 | -0.284 | -0.3 |
|  | Sub12 | -0.106 | -0.24 | 0.118 | -0.725** | -0.4 | -0.278 | 0.15 | 0.064 | -0.087 |
|  | Sub13 | -0.122 | -0.164 | 0.069 | -0.763** | -0.395 | -0.015 | -0.07 | -0.217 | -0.038 |
|  | Sub15 | -0.309 | -0.096 | 0.355 | -0.293 | -0.2 | -0.171 | 0.711** | 0.149 | -0.284 |
|  | Sub17 | 0.042 | 0.402 | 0.741** | 0.48 | 0.612* | -0.007 | 0.224 | 0.229 | 0.119 |
|  | Sub18 | -0.191 | -0.422 | 0.354 | -0.055 | 0.710** | -0.465 | 0.474 | 0.356 | -0.228 |
|  | Sub20 | -0.616* | -0.571* | 0.701* | 0.333 | 0.097 | 0 | 0.469 | 0.202 | -0.653* |
|  | Sub24 | -0.836** | -0.462 | 0.462 | -0.654* | -0.187 | 0.095 | 0.174 | -0.104 | -0.647* |
|  | Sub25 | -0.264 | -0.488 | 0.446 | -0.187 | -0.356 | 0.183 | 0.608* | 0.232 | -0.304 |
|  | Sub26 | -0.647* | -0.236 | 0.655* | 0.573* | 0.563 | -0.241 | 0.201 | 0.212 | -0.657* |

Significant levels for the Spearman’s rank coefficients are indicated at the *P<0.05; **P<0.01 level.

The same below.

Table S6 Relationships between the relative abundance of *Acidobacteria* subgroups (subsoil) and environment factors (subsoil) by Spearman's rank correlation coefficient test

|  | | Environmental factors | | | | | | | | |
| --- | --- | --- | --- | --- | --- | --- | --- | --- | --- | --- |
|  |  | OM | NO_3_^-^-N | SM | pH | H’_Vegetation_ | D’_Vegetation_ | J’_Vegetation_ | DBH | Hight |
| *Acidobacteria* subgroups | Sub1 | 0.849** | 0.212 | 0.002 | -0.772** | 0.161 | -0.196 | -0.301 | -0.35 | 0.245 |
|  | Sub2 | 0.497 | -0.242 | -0.4 | -0.793** | 0.133 | -0.266 | -0.035 | -0.476 | -0.049 |
|  | Sub3 | 0.607* | -0.547 | -0.506 | 0.025 | 0.602* | -0.559 | 0.343 | -0.594* | -0.294 |
|  | Sub4 | -0.630* | -0.28 | -0.277 | 0.803** | 0.007 | 0.168 | -0.063 | 0.524 | 0.182 |
|  | Sub5 | -0.148 | -0.571 | -0.08 | 0.572 | 0.056 | -0.112 | -0.154 | -0.469 | 0.168 |
|  | Sub6 | -0.488 | -0.366 | -0.125 | 0.811** | -0.287 | 0.266 | 0.322 | 0.252 | -0.217 |
|  | Sub7 | -0.383 | -0.186 | 0.119 | 0.689* | -0.294 | -0.392 | 0.776** | -0.014 | -0.559 |
|  | Sub10 | -0.577* | -0.731** | -0.315 | 0.621* | 0.392 | -0.434 | 0.042 | 0.063 | -0.07 |
|  | Sub11 | 0.295 | -0.361 | -0.491 | 0.212* | 0.147 | 0.028 | -0.28 | -0.203 | 0.203 |
|  | Sub12 | 0.258 | -0.115 | 0.122 | 0.21 | 0.221 | -0.178 | -0.37 | -0.007 | 0.299 |
|  | Sub13 | -0.19 | -0.593* | -0.739** | 0.061 | 0.343 | -0.098 | -0.28 | -0.476 | 0.098 |
|  | Sub15 | -0.148 | -0.152 | 0.195 | 0.592* | -0.294 | -0.357 | 0.028 | -0.497 | 0.014 |
|  | Sub17 | -0.472 | -0.193 | -0.454 | 0.768** | -0.133 | 0.168 | -0.014 | 0.343 | 0.07 |
|  | Sub18 | -0.651* | -0.011 | 0.032 | 0.832** | -0.084 | 0.168 | 0.133 | -0.021 | -0.168 |
|  | Sub20 | -0.582* | 0.051 | 0.617* | 0.22 | -0.092 | -0.137 | 0.046 | 0.062 | -0.054 |
|  | Sub24 | -0.3 | -0.675* | -0.338 | 0.655* | 0.364 | -0.322 | -0.007 | -0.126 | -0.161 |
|  | Sub25 | -0.175 | -0.298 | -0.197 | 0.700* | 0.049 | -0.063 | 0.049 | 0.21 | 0.063 |
|  | Sub26 | -0.561 | -0.032 | 0.238 | 0.751** | -0.176 | 0.113 | 0.211 | -0.155 | -0.359 |

Figure S1 Location of the soil samples in Pingquan County, Hebei province, China. H, high severity; M, moderate severity; L, low severity; C, unburnt control


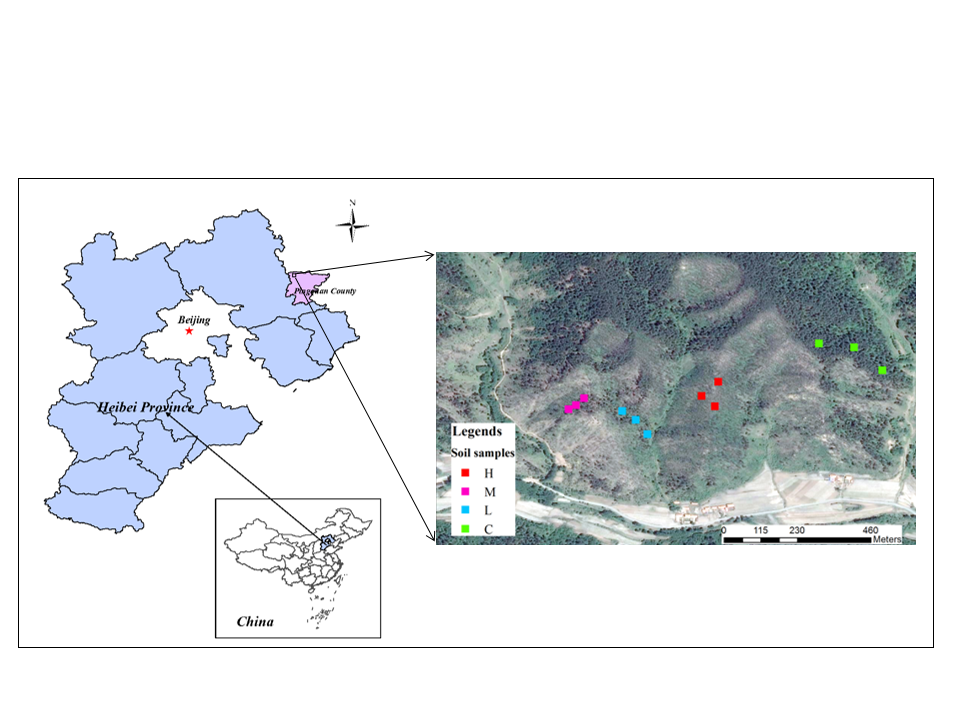


Figure S2 Diversity indices of understory vegetation after different fire severities.





Figure S3 the relative abundance of the dominant phyla (abundance >5%) across total soil samples.

**

**

Figure S4 Non-metric multidimensional scaling (NMDS) analysis based on Bray-Curtis distance (a) and Weighted-Unifrac distance (b) showing differences in Acidobacterial community structure.


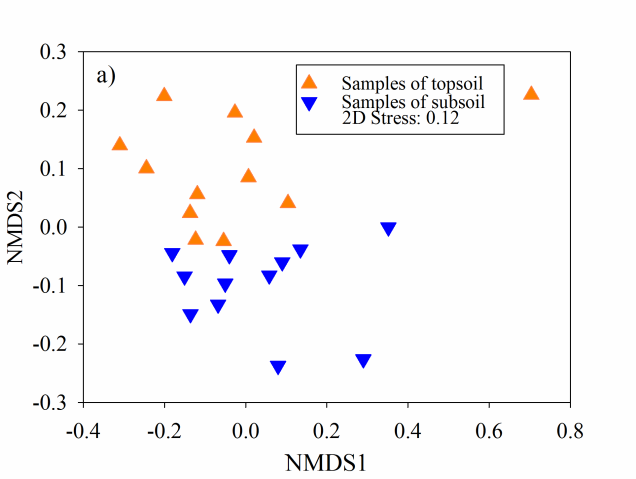

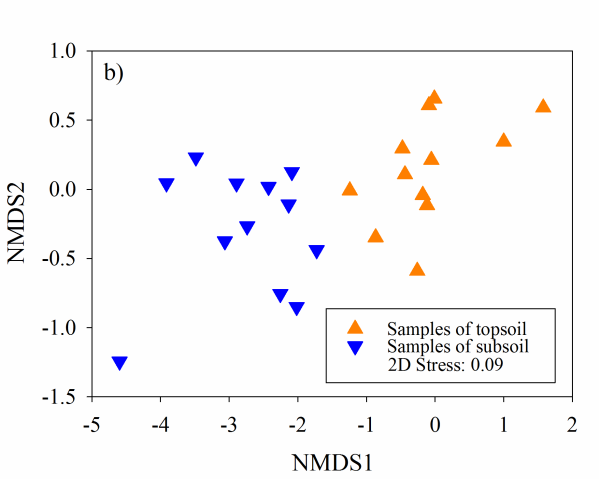

Supplement: Supplemental Information 1 [file peerj-07-8047-s001.docx]
